# Integrated Physiological and Transcriptomic Profiling of Tall Fescue in Response to Low-Nitrogen Stress

**DOI:** 10.3390/plants15213261

**Published:** 2026-10-22

**Authors:** Tian Hao, Jingjin Yu, Qiuguo Li, Ningli Fan, Binbin Liu, Jiayue Liang, Zhimin Yang

**Affiliations:** College of Agro-Grassland Science, Nanjing Agricultural University, Nanjing 210095, China; 2021220007@stu.njau.edu.cn (T.H.); nauyjj@njau.edu.cn (J.Y.); 2023220009@stu.njau.edu.cn (Q.L.); 2018220003@njau.edu.cn (N.F.); 2025220003@stu.njau.edu.cn (B.L.); 2025120018@stu.njau.edu.cn (J.L.)

**Keywords:** tall fescue, low-nitrogen stress, transcriptomic analysis, carbon and nitrogen metabolism, transcription factors, expression patterns

## Abstract

Low nitrogen (LN) availability is a major constraint limiting plant vegetative growth and reproductive development. To investigate the physiological and molecular mechanisms underlying LN stress in tall fescue (*Festuca arundinacea*), a comprehensive analysis of phenotypic, physiological, and transcriptional responses was conducted. The results showed that LN stress significantly reduced shoot height, tiller number, leaf length, and shoot biomass. In addition, root system architecture was markedly altered, with increased total root length but decreased root number and root volume. Physiological assays revealed that total chlorophyll content, photochemical efficiency, and net photosynthetic rate were significantly reduced. Concurrently, nitrate reductase activity, NO_3_^−^ concentration, and N content were significantly reduced, while soluble sugar content was significantly enhanced. Transcriptomic analysis identified a total of 1185 differential expression genes (585 upregulated and 600 downregulated), with significant changes in genes involved in N transport, N assimilation, glycolysis, and the tricarboxylic acid cycle. In addition, 27 transcription factor genes across seven distinct families were significantly differentially expressed. Three bHLH transcription factors, *FaIRO2*, *FabHLH69*, and *FabHLH062*, were identified as candidate regulators of the LN response in tall fescue based on their expression patterns under LN stress. Collectively, this study provides integrated physiological and transcriptional insights into tall fescue responses to LN stress and highlights potential target genes for genetic improvement of perennial forage and turf grasses with high N use efficiency.

## 1. Introduction

Plant growth and development is influenced by various factors, including light, water, and mineral nutrients [1,2]. Among the macronutrients required by plants, nitrogen (N) is an essential macronutrient for plants and a key component of amino acids, nucleic acids, chlorophyll (Chl), and various secondary metabolites [3]. These organic N compounds play essential roles in photosynthesis, carbon (C) and N metabolism, energy transfer, signal transduction, and growth and development regulation. Except for a few plants that can directly utilize atmospheric N_2_ through symbiotic relationships with soil bacteria, most plants acquire N from the soil [4]. The forms of N taken up by plants from the soil include inorganic N (NH_4_^+^ and NO_3_^−^) and a range of low-molecular-weight organic N compounds such as amino acids, oligopeptides, and amino sugars, and nitrate (NO_3_^−^) is the main form taken up and assimilated by most plants [5]. However, NO_3_^−^ is highly mobile and readily lost from the soils through leaching and denitrification, and to a lesser extent through runoff [6]. Consequently, soils are often deficient in NO_3_^−^, resulting in low-nitrogen (LN) stress.

Low-nitrogen stress reduces plant photosynthetic capacity by affecting the synthesis of photosynthetic pigments, the efficiency of the photosynthetic electron transport chain, and the activities of key enzymes involved in C fixation [7]. Concurrently, it leads to an imbalance in C and N metabolism and impairs protein synthesis, ultimately resulting in weak plant growth, leaf chlorosis, reduced tillering, and decreased biomass. In agricultural production systems, crop yield and quality depend on substantial N input. LN inhibits the growth of diverse crops such as barley (*Hordeum vulgare*), maize (*Zea mays*), rice (*Oryza sativa*), and sorghum (*Sorghum bicolor*), manifesting as reduced plant height, fewer tillers, and lower yield [8,9,10,11]. Under LN stress, plants have evolved complex adaptive mechanisms to maintain N uptake and utilization efficiency as well as internal N homeostasis. These strategies primarily include modulating root system architecture, optimizing C-N allocation ratios, inducing the expression of high-affinity N transporter genes such as NRT2 and AMT families, enhancing the activities of key N-assimilating enzymes, and mobilizing endogenous N for remobilization [12,13].

Studies have shown that under LN stress, auxin levels at lateral root tips decrease, leading to inhibition of lateral root elongation [14]. However, plants often enhance NO_3_^−^ uptake by increasing biomass or root length. NO_3_^−^ absorbed by roots must undergo long-distance transport to reach various shoot organs, a process that mainly relies on nitrate transporters. In higher plants, the NO_3_^−^ transport system comprises four families of transporters: nitrate transporter 1/peptide transporter (NRT1/NPF), nitrate transporter 2 (NRT2), the chloride channel (CLC), and slow anion channel-associated (SLAC)/SLAC1 homolog (SLAH) channels [15]. In addition to N uptake and transport, crop N use efficiency is also associated with N assimilation. N assimilation refers to the metabolic process by which plants convert absorbed inorganic N (NO_3_^−^ and NH_4_^+^) into organic N compounds through a series of enzymatic reactions [16]. NR catalyzes the reduction of NO_3_^−^ to NO_2_^−^ in the cytoplasm. The resulting NO_2_^−^ is subsequently transported into plastids, where it is further reduced to NH_4_^+^ by nitrite reductase (NiR). NH_4_^+^ is ultimately assimilated through the GS/GOGAT cycle, converting inorganic N into organic N [17]. N assimilation depends on C skeletons and reducing power generated by photosynthesis, while photosynthesis depends on N invested in Chl, enzymes, and structural components of the photosynthetic machinery [18]. Therefore, these two processes are interdependent and must be coordinately regulated. Such coordination relies on transcriptional reprogramming, in which transcription factors (TFs) act as central hubs integrating NO_3_^−^ signals with cellular C-N status. Under LN stress, multiple TFs coordinately regulate the expression of NO_3_^−^ transporters and assimilation genes, thereby adjusting N uptake, assimilation, and C allocation to maintain C-N balance and confer LN tolerance [19,20].

Although many studies have explored the mechanisms underlying plant tolerance to LN stress, most studies have focused on staple food crops such as rice, maize, and wheat [9,10,21]. The amount and frequency of N fertilizer application are directly related to forage production and turf quality [22]. However, research on LN stress responses in forage and turf grasses remains relatively limited. Moreover, research that concurrently examines the response of grasses to LN stress from both shoot and root perspectives, while also considering C and N metabolic pathways, remains scarce. Tall fescue (*Festuca arundinacea*) is a commonly used perennial cool-season turfgrass and forage grass [23,24]. It exhibits several advantageous traits, including rapid growth rate, strong tillering ability, and high trampling tolerance, which render it widely used in forage production, landscaping, and ecological restoration [25]. Although previous studies have preliminarily elucidated the physiological responses of tall fescue to LN stress, the transcriptional regulatory networks remain poorly understood, and the key upstream regulatory genes and associated molecular pathways require further exploration. In this context, transcriptome sequencing enables systematic characterization of global gene expression changes under LN stress, thereby compensating for the limitations of previous mechanistic studies.

We hypothesize that LN stress alters root architecture, reduces photosynthetic capacity, and reprograms C-N metabolic pathways in tall fescue. These physiological adaptive processes are transcriptionally regulated by specific TFs, which participate in the LN stress responses by modulating the expression of downstream target genes. Accordingly, this study integrated physiological, biochemical, and transcriptomic analyses to elucidate the mechanisms underlying the responses of tall fescue shoots and roots to LN stress, with a focus on C and N metabolic pathways. The results of this study will provide a theoretical basis and candidate genes for breeding new grass varieties that are tolerant to LN stress, including forage and turf grasses.

## 2. Results

### 2.1. The Effects of LN Stress on Shoot Growth of Tall Fescue

**Figure 1:**
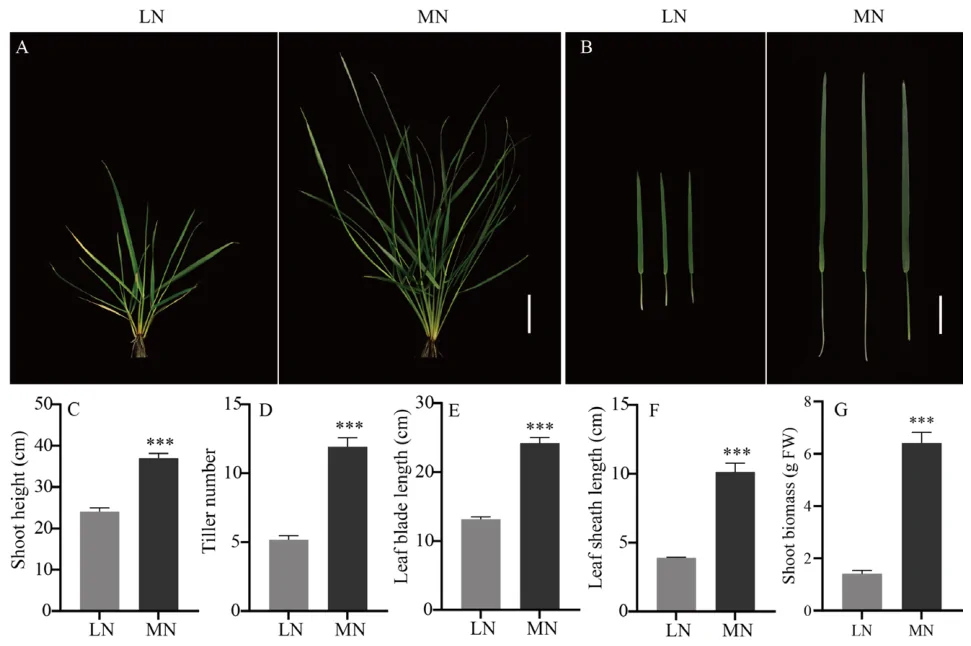
The effects of LN stress on shoot growth of tall fescue. Bar = 5 cm. (**A**) Shoot phenotype. (**B**) Leaf phenotype. (**C**) Shoot height. (**D**) Tiller number. (**E**) Leaf blade length. (**F**) Leaf sheath length. (**G**) Shoot biomass. Asterisks represent significant differences between LN and MN treatments by Student’s *t*-test. Significance level: *** *p* < 0.001. Error bars represent standard error (*n* = 4).

To investigate the effects of LN on the growth of tall fescue, 21-day-old seedlings were exposed to treatment solutions containing LN or moderate nitrogen (MN) for 35 d. The results showed that LN stress suppressed shoot growth, with significant reductions in shoot height, tiller number, leaf blade length, leaf sheath length, and shoot biomass by 34.9% (Figure 1C), 56.6% (Figure 1D), 45.8% (Figure 1E), 61.6% (Figure 1F), and 77.9% (Figure 1G), respectively, compared with MN.

### 2.2. The Effects of LN Stress on Root Growth of Tall Fescue

**Figure 2:**
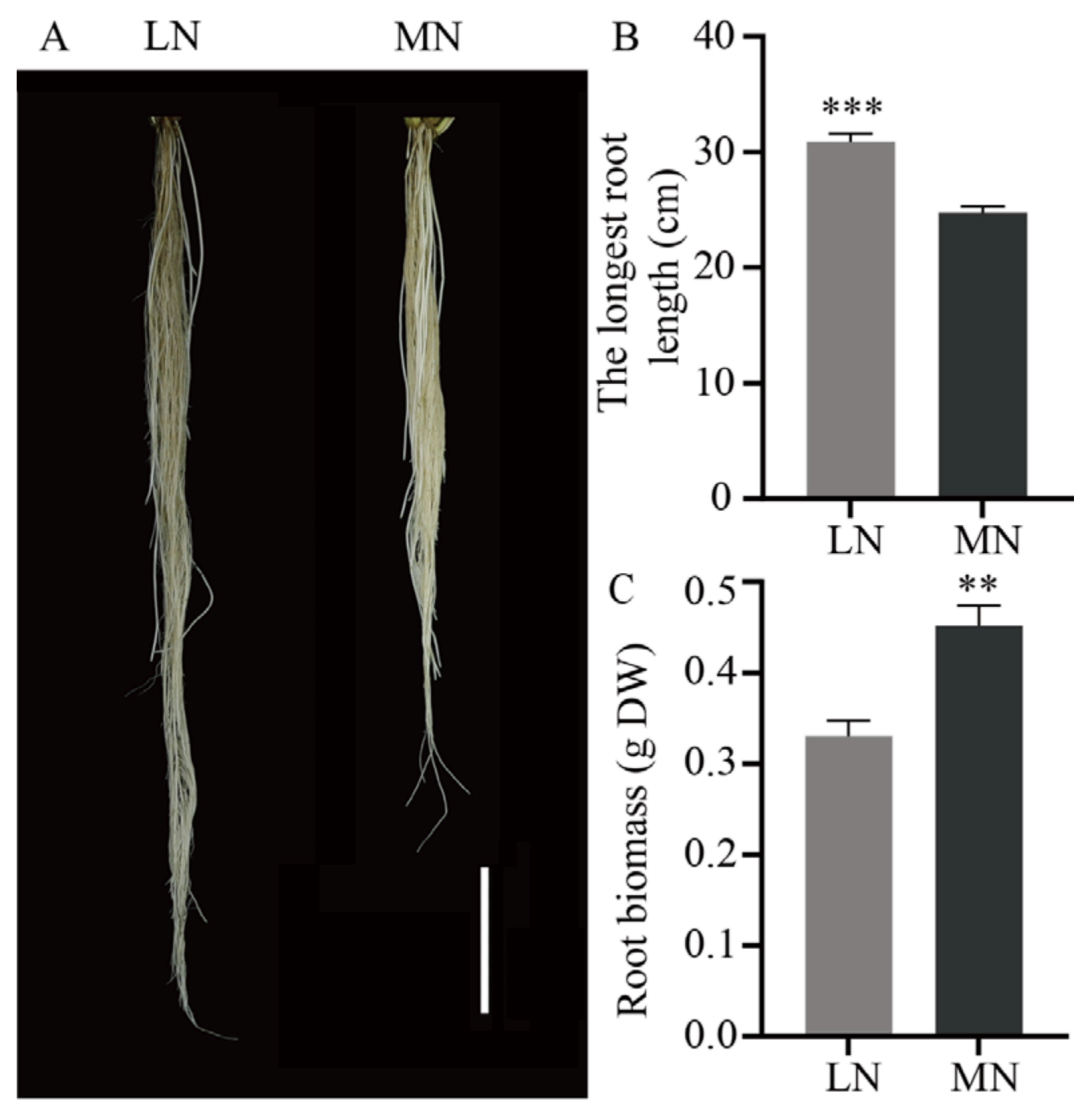
The effects of LN stress on root growth of tall fescue. Bar = 5 cm. (**A**) Root phenotype. (**B**) The longest root length. (**C**) Root biomass. Asterisks represent significant differences between LN and MN treatments by Student’s *t*-test. Significance level: ** *p* < 0.01, *** *p* < 0.001. Error bars represent standard error (*n* = 4).

Low-nitrogen stress promoted root elongation but inhibited root biomass in tall fescue. Compared with MN, the longest root length increased significantly by 24.7% (Figure 2B), while root biomass decreased significantly by 26.9% (Figure 2C).

**Figure 3:**
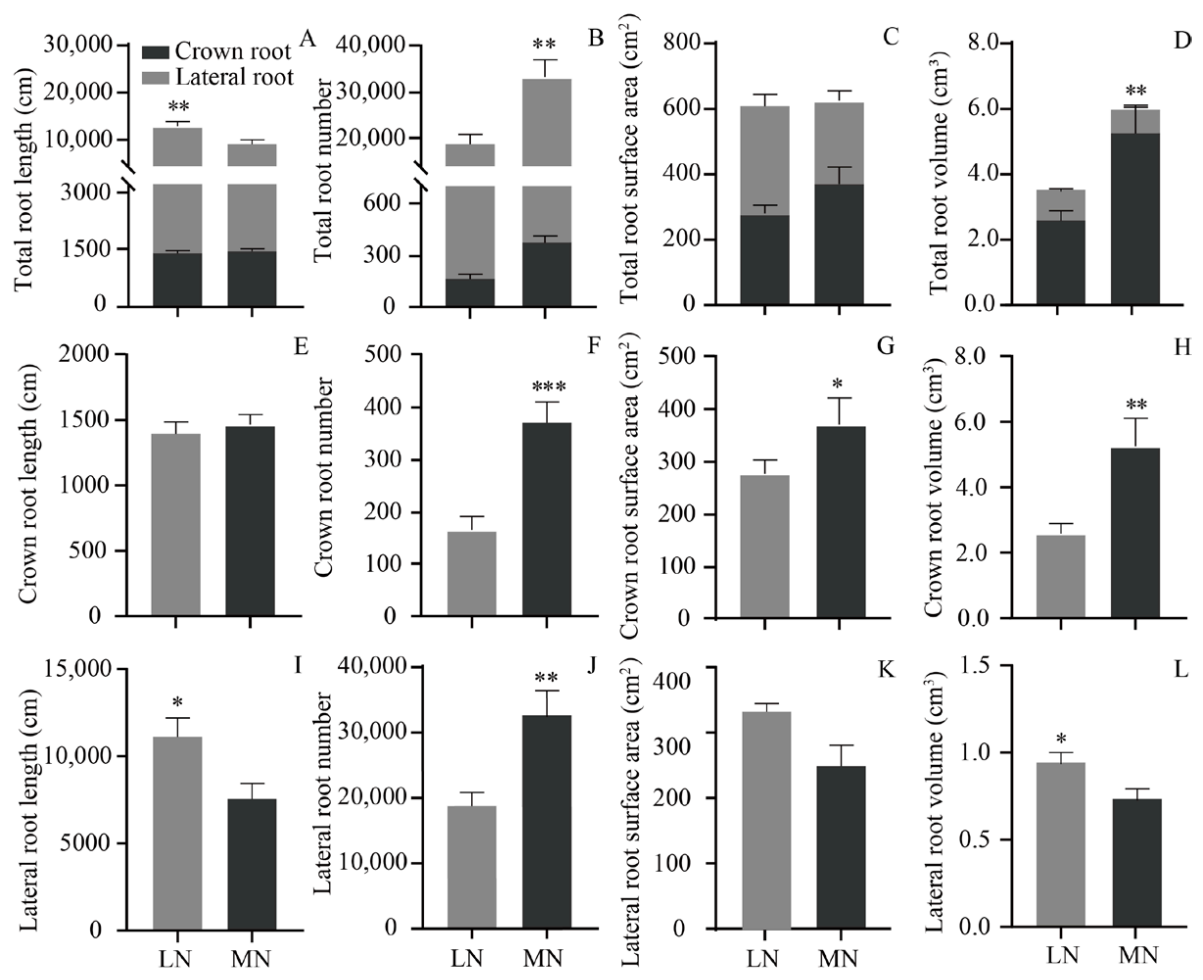
The effects of LN stress on root morphological parameters of tall fescue. (**A**) Total root length. (**B**) Total root number. (**C**) Total root surface area. (**D**) Total root volume. (**E**) Crown root length. (**F**) Crown root number. (**G**) Crown root surface area. (**H**) Crown root volume. (**I**) Lateral root length. (**J**) Lateral root number. (**K**) Lateral root surface area. (**L**) Lateral root volume. (**A**–**D**). Stacked bars represent the sum of crown root (black) and lateral root (gray) components, with the total height of each bar indicating the combined value. (**E**–**L**) Bars represent independent measurements of crown or lateral roots. Asterisks represent significant differences between LN and MN treatments by Student’s *t*-test. Significance level: * *p* < 0.05, ** *p* < 0.01, *** *p* < 0.001. Error bars represent standard error (*n* = 4).

To investigate the effects of LN on root architecture, roots were scanned with a root scanner and analyzed. The results showed that, compared with MN, LN suppressed the number (Figure 3F), surface area (Figure 3G), and volume of crown roots by 56.1%, 25.6% and 50.9%, respectively but had no significant effect on their length (Figure 3E,H). LN significantly reduced the number of lateral roots by 42.4% (Figure 3J) and increased their length and volume by 47.1% and 28.3% (Figure 3I,L) but had no significant effect on their surface area (Figure 3K). Furthermore, under LN stress, total root length increased by 68.7% (Figure 3A), while total root number and volume decreased by 42.6% and 40.0%, respectively (Figure 3B,D).

### 2.3. The Effects of LN Stress on Photosynthetic Performance of Tall Fescue

**Figure 4:**
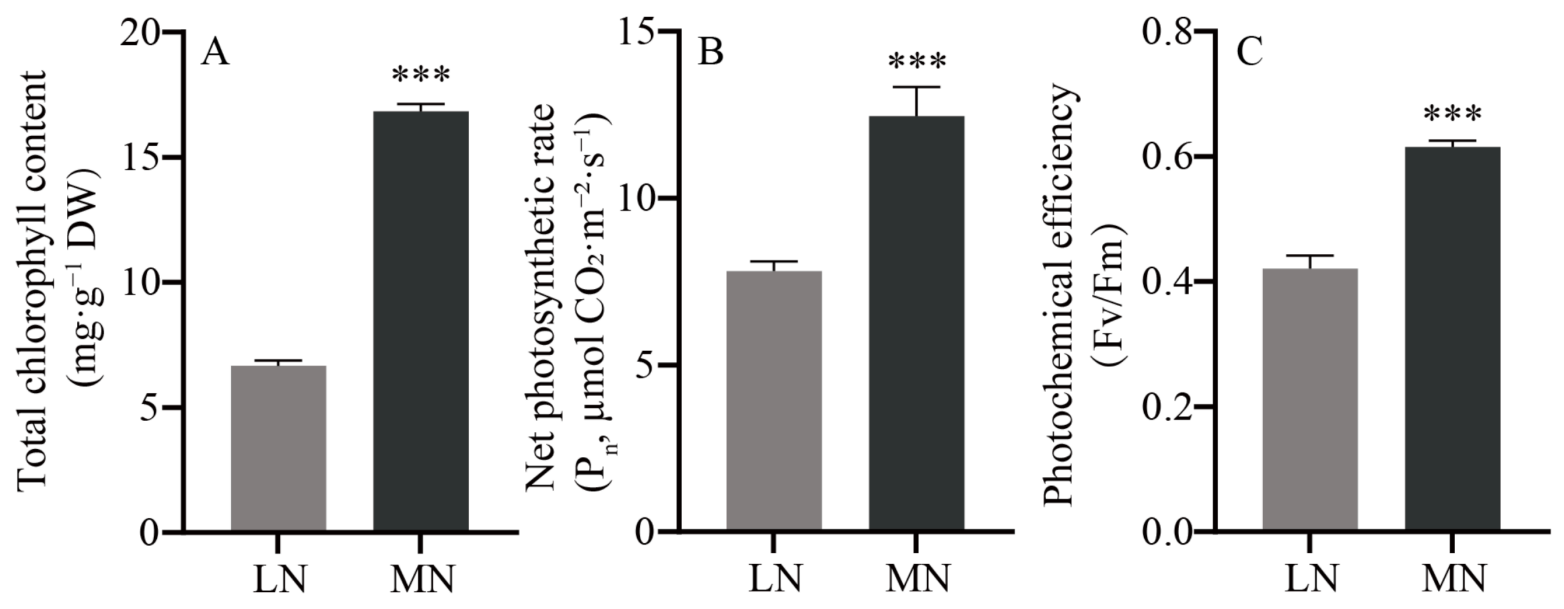
The effects of LN stress on photosynthetic performance of tall fescue. (**A**) Total chlorophyll content. (**B**) Net photosynthetic rate. (**C**) Photochemical efficiency. Asterisks represent significant differences between LN and MN treatments by Student’s *t*-test. Significance level: *** *p* < 0.001. Error bars represent standard error (*n* = 3).

To investigate the effects of LN on photosynthetic performance of tall fescue, total Chl content, photosynthetic rate (P_n_), and photochemical efficiency (F_v_/F_m_) were measured. As shown in Figure 4, LN stress significantly reduced total Chl content, P_n_, and F_v_/F_m_ by 60.4%, 37.2%, and 31.6%, respectively, compared with MN.

### 2.4. The Effects of LN Stress on N Metabolism of Tall Fescue

**Figure 5:**
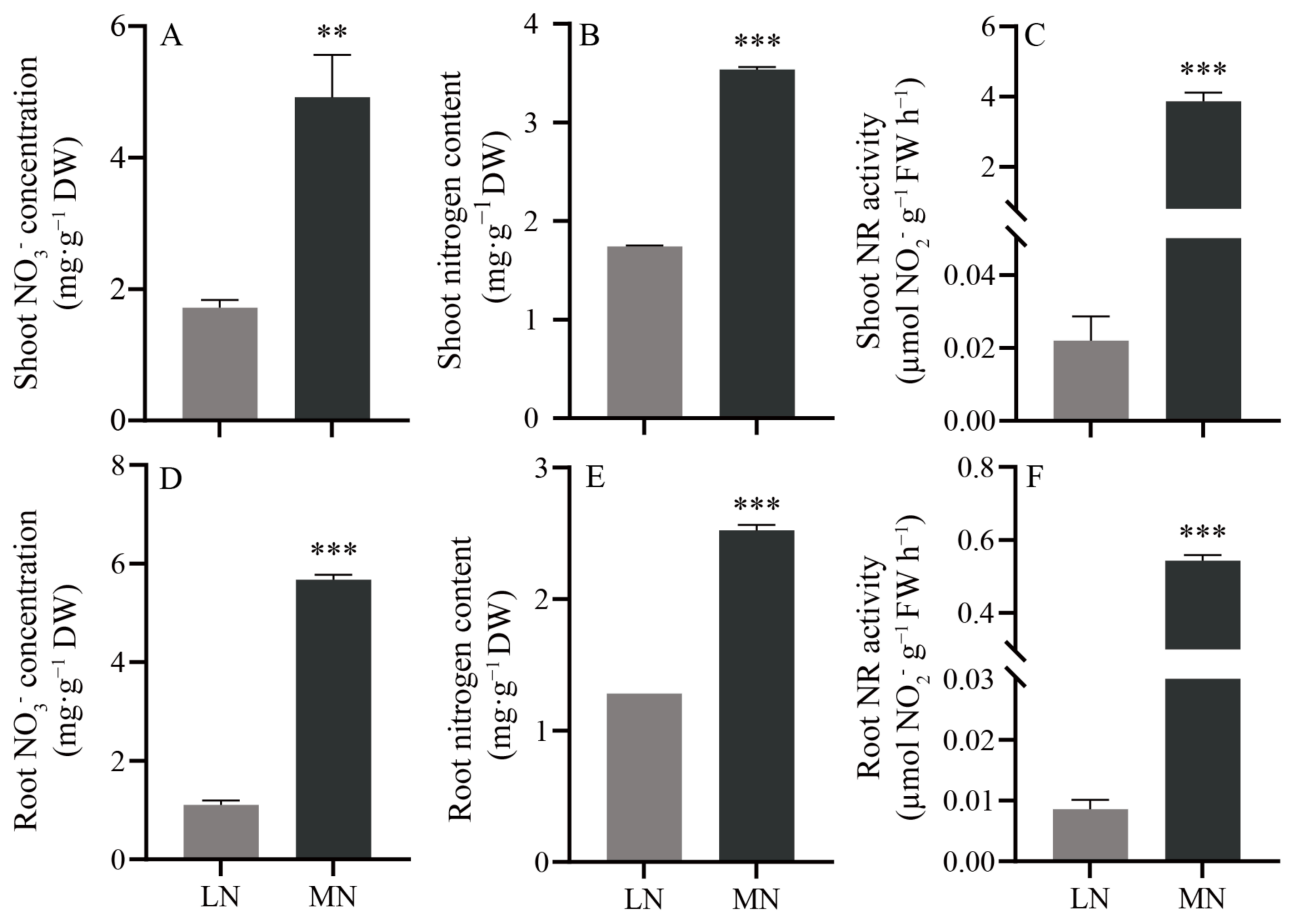
The effects of LN stress on nitrogen metabolism of tall fescue. (**A**) Shoot NO_3_^−^ concentration. (**B**) Shoot nitrogen content. (**C**) Shoot nitrate reductase activity (broken *y*-axis). (**D**) Root NO_3_^−^ concentration. (**E**) Root nitrogen content. (**F**) Root nitrate reductase activity (broken *y*-axis). The y-axes in (**C**,**F**) are broken to accommodate the large differences between LN and MN treatments. Asterisks represent significant differences between LN and MN treatments by Student’s *t*-test. Significance level: ** *p* < 0.01, *** *p* < 0.001. Error bars represent standard error (*n* = 3).

To investigate the effects of LN on N metabolism in tall fescue, NO_3_^−^ concentration, N content, and nitrate reductase (NR) activity in both shoots and roots were measured after treatments. In shoots, under LN stress, NO_3_^−^ concentration, N content, and NR activity were reduced by 65.1%, 50.7%, and 99.4%, respectively, compared with MN (Figure 5A–C). In roots, LN stress caused a significant reduction in NO_3_^−^ concentration, N content, and NR activity by 80.5%, 49.1%, and 98.4%, respectively, compared with MN (Figure 5D–F).

### 2.5. The Effects of LN Stress on Soluble Sugar Content of Tall Fescue

**Figure 6:**
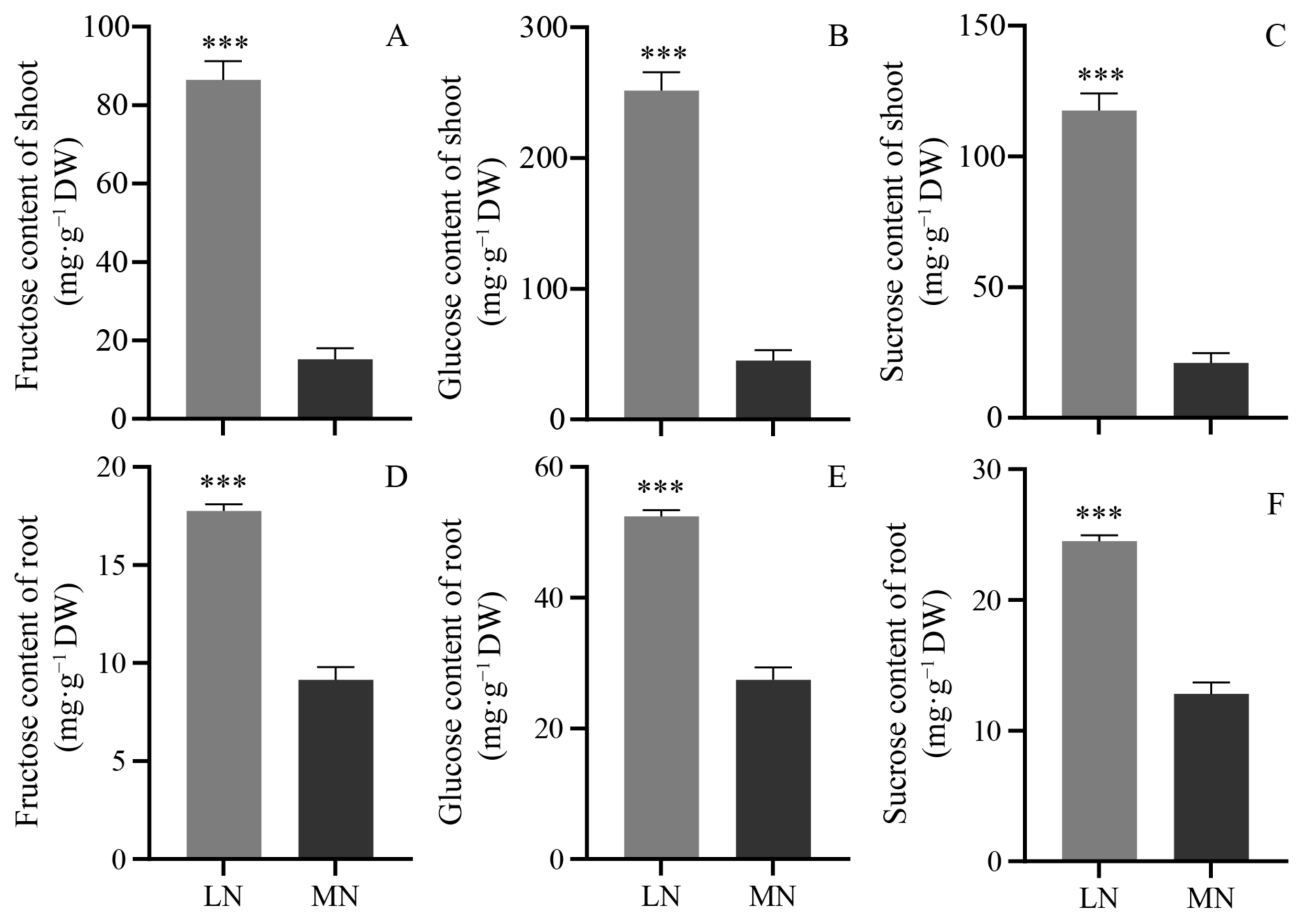
The effects of LN stress on soluble sugar content of tall fescue. (**A**) Fructose content of shoot. (**B**) Glucose content of shoot. (**C**) Sucrose content of shoot. (**D**) Fructose content of root. (**E**) Glucose content of root. (**F**) Sucrose content of root. Asterisks represent significant differences between LN and MN treatments by Student’s *t*-test. Significance level: *** *p* < 0.001. Error bars represent standard error (*n* = 4).

To investigate the effects of LN on soluble sugar accumulation in tall fescue, the contents of glucose, sucrose, and fructose in both shoots and roots were measured. In shoots, LN stress increased glucose, sucrose, and fructose contents to similar extents, with an average increase of 462.8% compared with MN (Figure 6A–C). In roots, the average increase across the three sugars was approximately 92.1% compared with MN (Figure 6D–F).

### 2.6. Analysis of Differential Expression Genes (DEGs)

**Figure 7:**
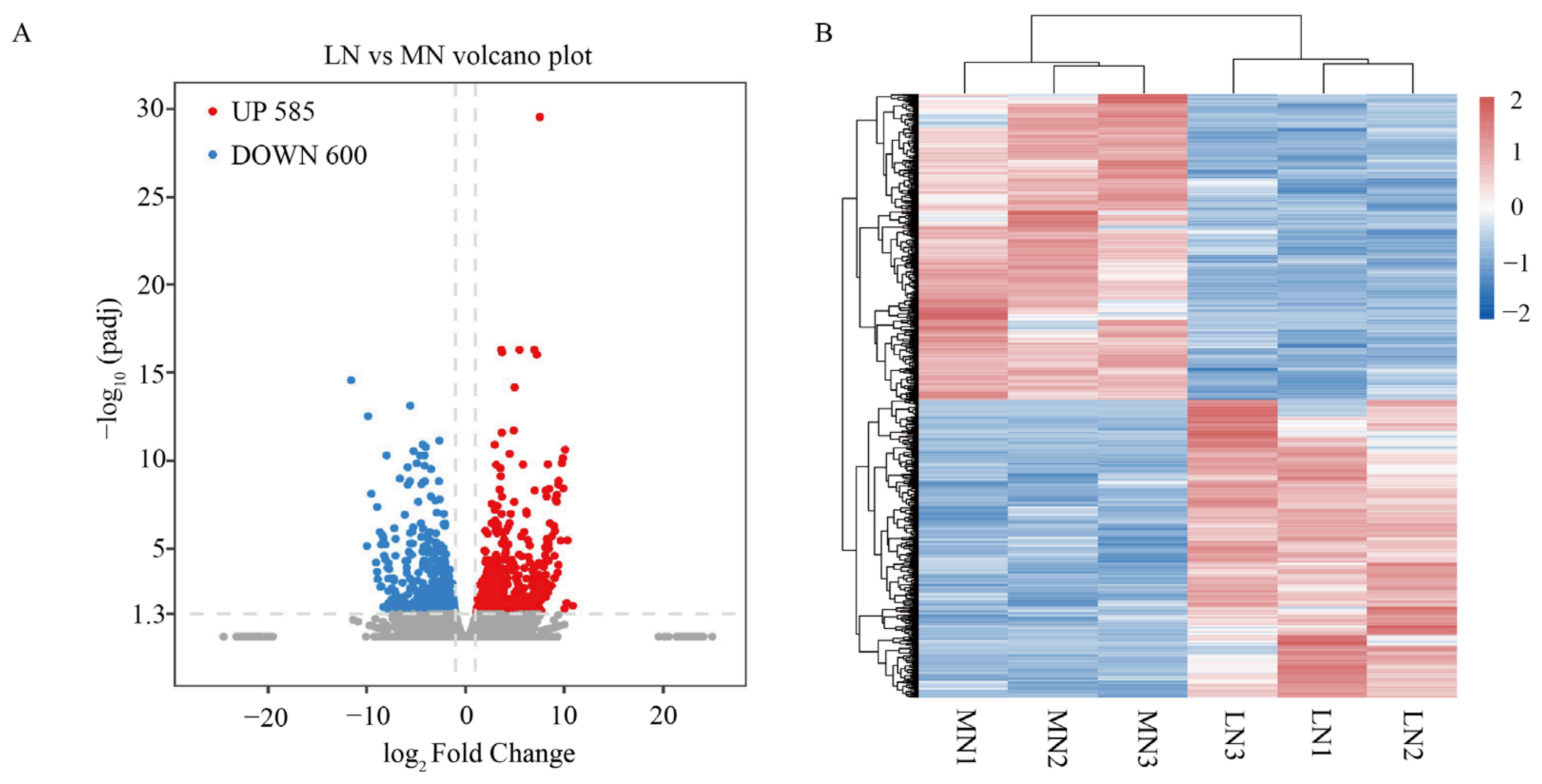
Transcriptomic analysis of LN and MN treatments. (**A**) Volcano plots of differentially expressed genes between LN and MN treatments. (**B**) Heatmap of differentially expressed genes clustering between LN and MN treatments. Three biological replicates per treatment were used (*n* = 3).

Transcriptomic sequencing of LN and MN plants, with three biological replicates per treatment, generated over 42 million clean reads. The proportion of bases with a Phred quality score ≥30 exceeded 97.18%, indicating high-quality RNA-sequencing (RNA-seq) data (Table S1). DEGs were identified using DESeq2 to identify DEGs between the LN and MN groups. DEGs were screened using the thresholds |log_2_ fold change| ≥ 1 and adjusted *p*-value (*P*adj) ≤ 0.05, and the results were visualized. The volcano plot revealed that 585 genes were significantly upregulated, accounting for 49.4% of the total DEGs, while 600 genes were significantly downregulated, accounting for 50.6% (Figure 7A). In addition, the heatmap revealed distinct expression patterns of DEGs between the LN and MN treatments (Figure 7B). Principal component analysis (PCA) of the transcriptomic data showed that the biological replicates were highly similar within each treatment, while the LN samples were clearly separated from the MN group (Figure S1A). Pearson correlation analysis revealed high reproducibility among the biological replicates (Figure S1B).

### 2.7. GO and KEGG Enrichment Analysis of DEGs

**Figure 8:**
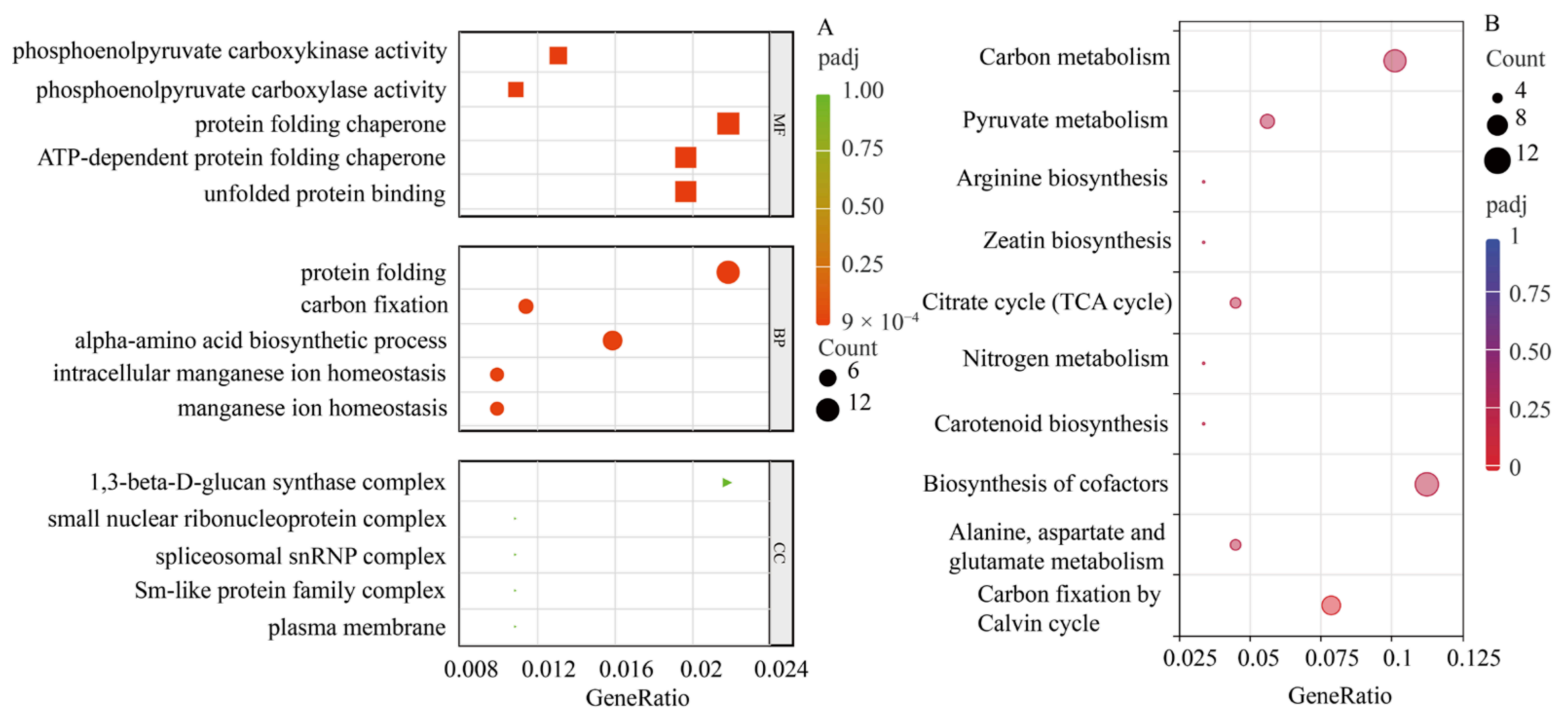
Analysis of differentially expressed genes between LN and MN treatments. (**A**) GO functional enrichment analysis. (**B**) KEGG functional enrichment analysis. The *Y*-axis shows the GO terms and KEGG pathways. The *X*-axis represents the GeneRatio. Dot size indicates the number of DEGs (Count), and dot color represents the *P*adj, with red indicating higher statistical significance.

To investigate the functions of the 1185 DEGs under LN stress, GO enrichment analysis was performed, and the significantly enriched GO terms were categorized into biological process (BP), molecular function (MF), and cellular component (CC). In the BP category, the enriched GO terms were mainly related to protein folding, C fixation, and alpha-amino acid biosynthetic process, among others. In the MF category, the top three enriched GO terms were phosphoenolpyruvate carboxykinase activity, phosphoenolpyruvate carboxylase activity, and protein folding chaperone (Figure 8A).

The DEGs were further mapped to reference pathways in the KEGG database to elucidate key biological pathways involved in the response to LN stress. The top 10 pathways with the lowest *P*adj were selected for display. The top five KEGG pathways included C fixation by the Calvin cycle; alanine, aspartate, and glutamate metabolism; biosynthesis of cofactors; carotenoid biosynthesis; and N metabolism (Figure 8B).

### 2.8. The Expression Characteristics of Genes Related to C and N Metabolism in Response to Different N Treatments

**Figure 9:**
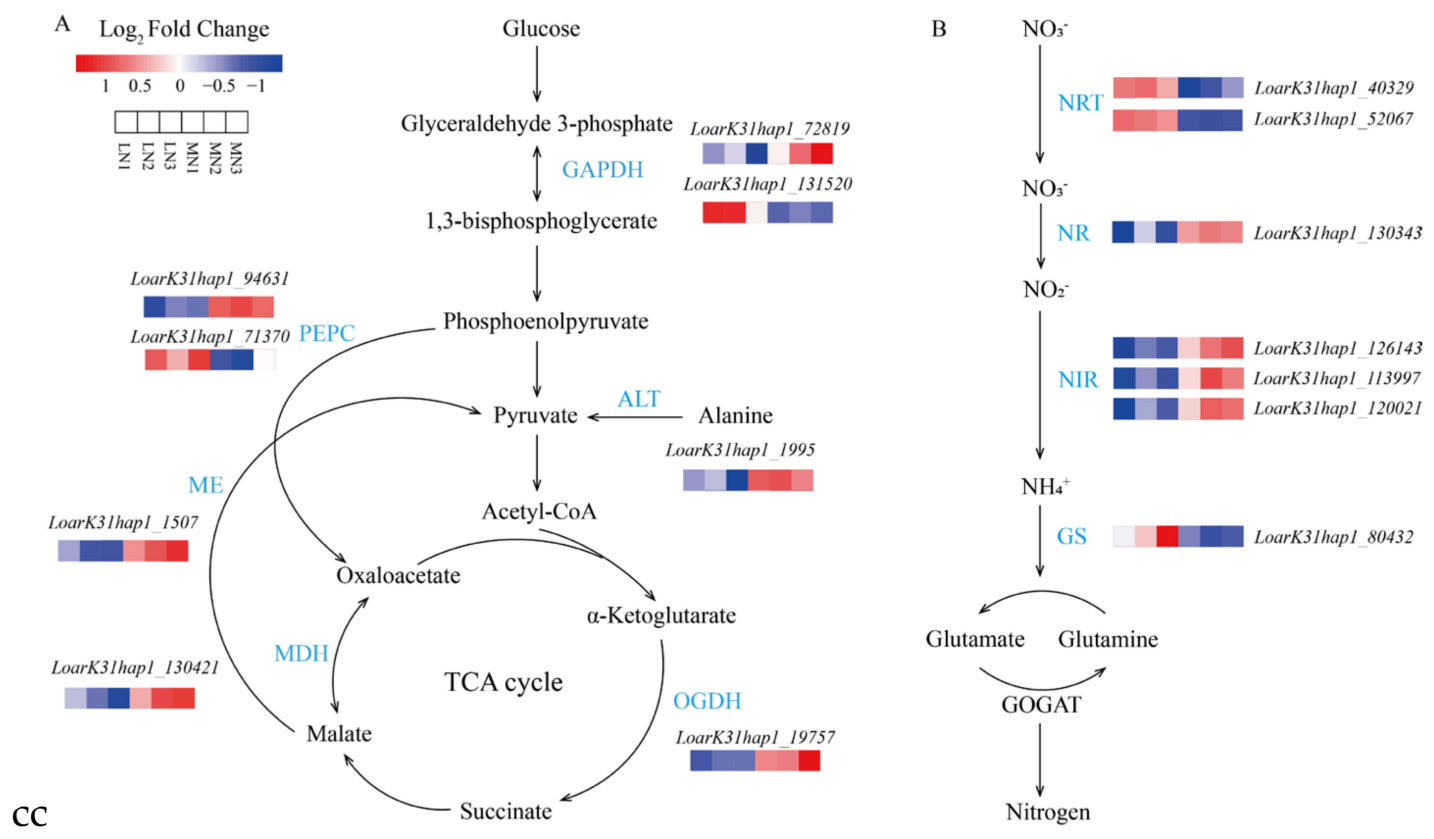
Expression analysis of differentially expressed genes related to carbon and nitrogen metabolism by RNA-Seq. (**A**) Expression patterns of genes in the carbon metabolism pathway. (**B**) Expression patterns of genes in the nitrogen metabolism pathway. Arrows indicate the direction of metabolic conversion, and double-headed arrows indicate reversible reactions. The heatmap colors represent log_2_ fold change (LN vs. MN), with red indicating upregulated expression, blue indicates downregulated expression, and white indicates no significant change. Enzyme names are indicated in blue.

Under LN stress, the expression of genes involved in C metabolism was affected. Several of these genes were differentially expressed, and the expression patterns of DEGs related to glycolysis and the tricarboxylic acid (TCA) cycle under LN and MN treatments were analyzed. Compared with MN, the expression levels of *GAPDH1* and *PEPC1* were significantly increased, whereas the expression of *ME*, *MDH*, *OGDH2*, *ALT*, *GAPDH2*, and *PEPC2* was significantly decreased due to LN stress (Figure 9A).

Under LN stress, the expression of genes involved in N uptake, translocation, and assimilation in tall fescue was significantly influenced. Several DEGs related to N translocation and assimilation were upregulated under LN stress, including genes encoding *NRT1*, *NRT2.4*, and *GS*, while genes encoding *NR* and *NIR* were significantly downregulated (Figure 9B).

### 2.9. The Expression of TFs Under LN Stress in Tall Fescue

**Figure 10:**
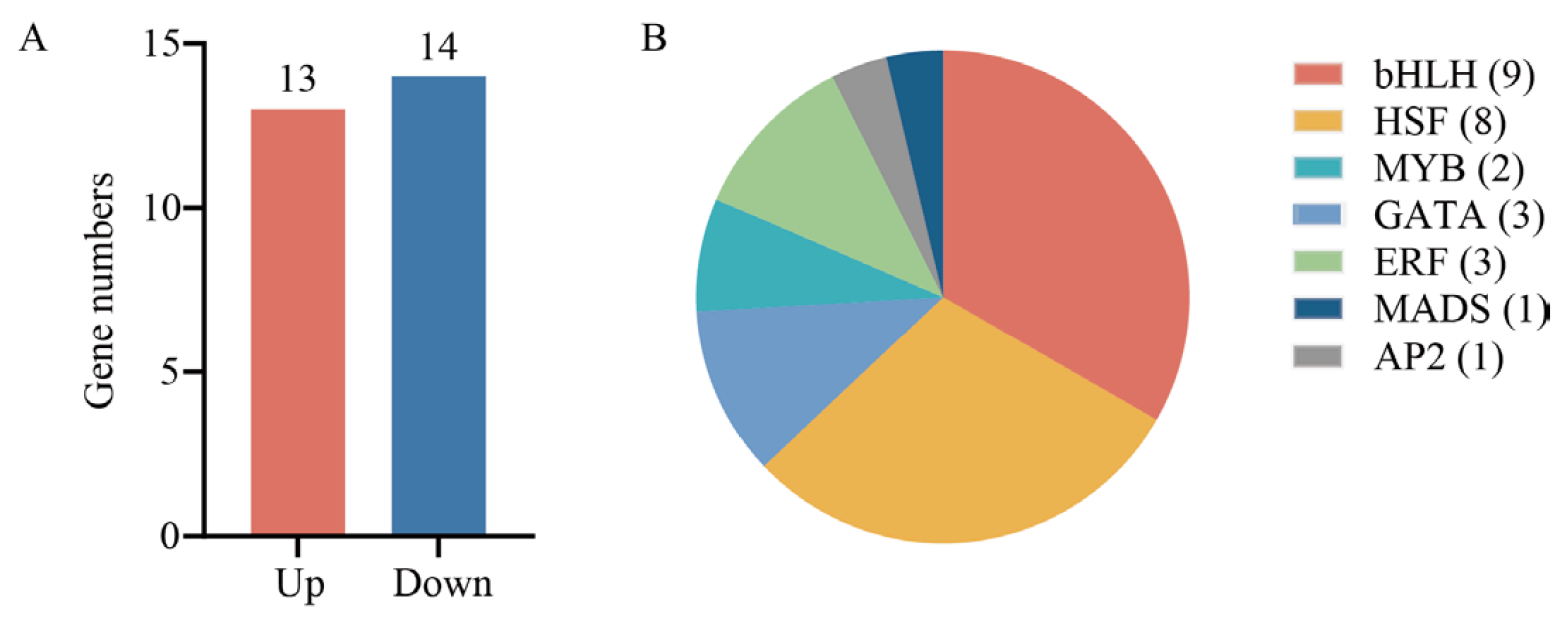
Transcription factor analysis of differentially expressed genes. (**A**) The number of upregulated and downregulated transcription factors between LN and MN treatments. (**B**) The family classification of the 27 transcription factors.

To elucidate the regulatory mechanisms of TFs, a comprehensive analysis of TFs in tall fescue was conducted under LN stress in this study. DEGs were screened with the thresholds |log_2_ fold change| ≥ 2 and *P*adj≤ 0.05, and the results revealed that 27 TFs belonging to seven families were differentially expressed (Figure 10). Among them, the bHLH TF family was the most abundant, with three genes upregulated and six downregulated, followed by the HSF TF family, with four genes upregulated and four downregulated.

### 2.10. Validation of TFs Temporal Expression Patterns of Under LN Stress by qRT-PCR

**Figure 11:**
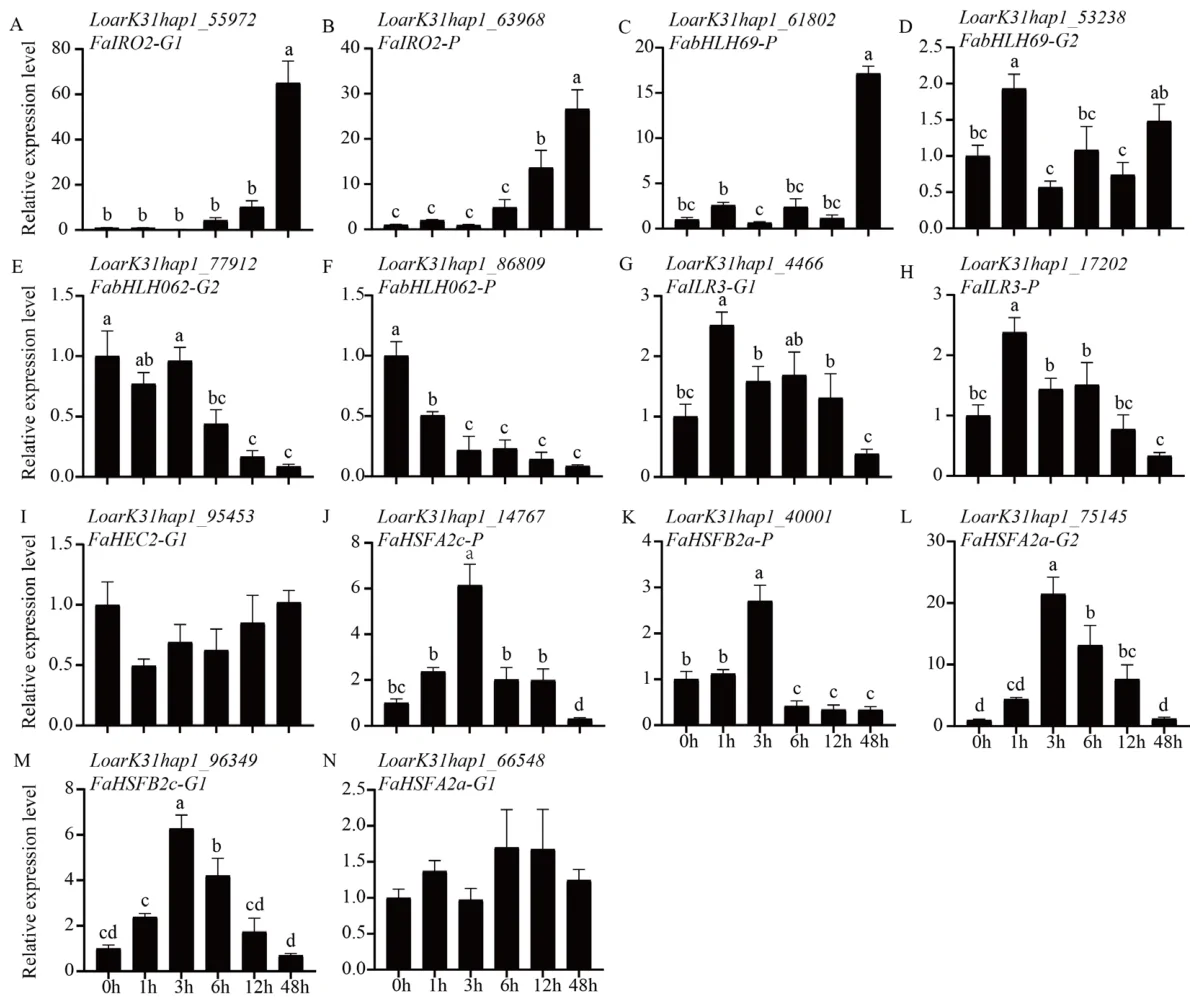
Temporal expression analysis of FabHLH and FaHSF family genes under LN stress in leaves. (**A**–**I**) bHLH transcription factors. (**J**–**N**) HSF transcription factors. Different letters indicate significant differences at *p* < 0.05 based on Duncan’s test. Error bars represent standard error (*n* = 3).

To further investigate the expression patterns of differentially expressed TFs in response to LN stress, the expression levels of nine bHLH TF genes and five HSF TF genes were analyzed at 0, 1, 3, 6, 12, and 48 h after LN stress. The results showed that each gene exhibited a distinct temporal expression pattern during LN stress. Based on their expression patterns, bHLH TF genes were classified into the following five types. The first type was the continuously upregulated type, including *FaIRO2-G1*, *FaIRO2-P*, and *FabHLH69-P* (Figure 11A–C). These genes showed relatively low expression at the beginning of LN stress and increased continuously with prolonged treatment, peaking at 48 h. The second type included genes that were not induced by LN, represented by *FaHEC2-G1* (Figure 11I). The third type was the continuously downregulated type, including *FabHLH062-G2* and *FabHLH062-P* (Figure 11E,F). These genes exhibited a gradual decrease in expression under continuous LN stress. The fourth type was the early upregulation type, including *FaILR3-G1* and *FaILR3-P* (Figure 11G,H). These genes showed relatively high expression at 1 h of LN stress and then gradually decreased with prolonged treatment. The fifth type was the fluctuating expression type, represented by *FabHLH69-G2* (Figure 11D). The expression of this gene fluctuated during LN stress without an obvious pattern.

HSF TF genes were classified into the following two types. The first type was the early upregulation type, including *FaHSFA2c-P*, *FaHSFA2a-P*, *FaHSFA2a-G2* and *FaHSFB2c-G1* (Figure 11J–M). These genes showed relatively high expression at 3 h of LN stress and then gradually decreased with prolonged treatment. The second type included genes that were not induced by LN, represented by *FaHSFA2a-G1* (Figure 11N).

### 2.11. Validation of DEGs by qRT-PCR

**Figure 12:**
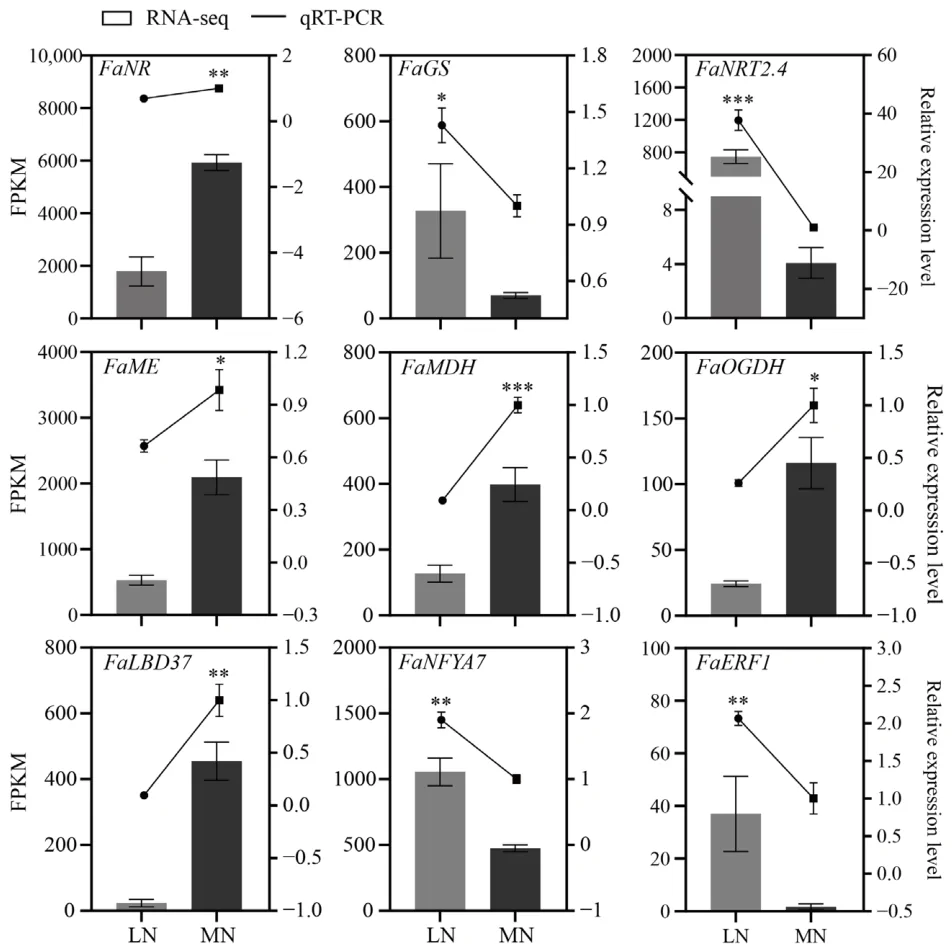
Validation of the transcription levels for the selected differentially expressed genes via qRT-PCR. RNA-Seq and qRT-PCR data are presented as bar and line graphs, respectively. * represents a significant difference between LN and MN treatments by Student’s *t*-test. Significance level: * *p* < 0.05, ** *p* < 0.01, *** *p* < 0.001. Error bars represent standard error (*n* = 3).

**Figure 13:**
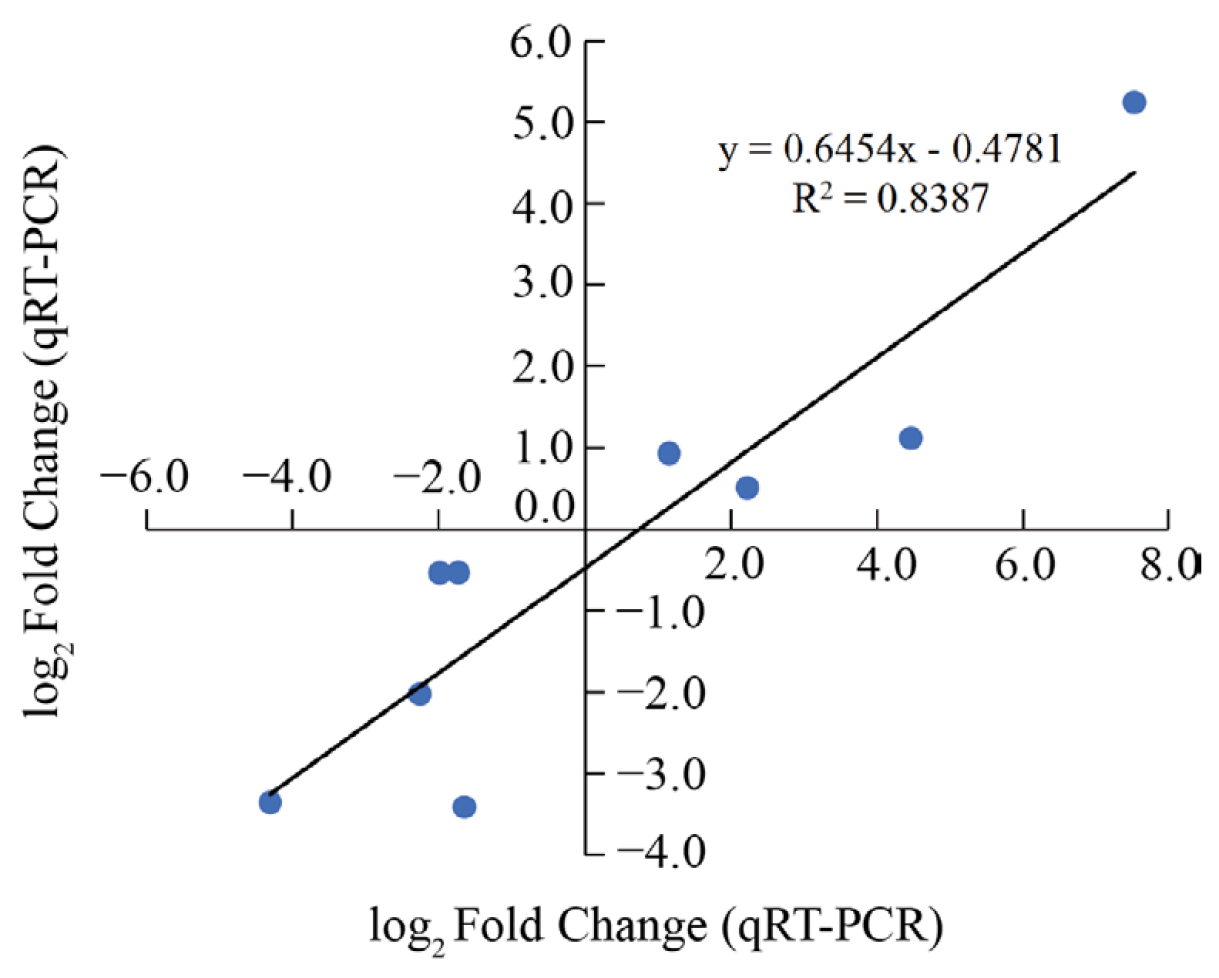
Comparison analysis between qRT-PCR and RNA-Seq data for nine differentially expressed genes under LN stress.

Six C-N metabolism genes and three TFs under LN and MN treatments were selected to validate the expression patterns obtained from the RNA-Seq data using qRT-PCR. The relative expression levels of *FaGS*, *FaNRT2.4*, *FaNFYA7*, and *FaERF1* were significantly upregulated, while those of *FaNR*, *FaME*, *FaMDH*, *FaOGDH*, and *FaLBD37* were downregulated under LN stress (Figure 12). For the nine DEGs validated by qRT-PCR, the log_2_ fold changes were highly correlated with those from RNA-seq (R^2^ = 0.8387), confirming the reliability of the RNA-seq data (Figure 13).

## 3. Discussion

Among the various nutrients required for plant growth, N is a major limiting factor that severely affects plant survival [26]. LN stress during plant growth significantly inhibits growth processes, including vegetative growth and reproductive development [10]. To mitigate the harm caused by LN stress, plants have evolved various adaptive mechanisms, including reduced biomass accumulation and photosynthesis, decreased N uptake, and modulation of shoot and root growth [27]. In this study, LN stress severely inhibited the shoot growth of tall fescue, resulting in significant reductions in shoot height, tiller number, leaf blade length, leaf sheath length, and fresh biomass, which is consistent with previous findings in tall fescue reported by Wen et al. [28]. In addition, LN altered root architecture. Both the longest root length and total root length were significantly greater under LN than MN, whereas total root number and root volume were significantly lower under LN stress (Figure 3). As the primary organ for N uptake, the root system exhibits strong plasticity. In response to heterogeneous N distribution in the soil resulting from processes such as leaching, plant roots exhibit rapid adaptive responses to maintain normal growth [29]. Root architecture involves the spatial distribution and expansion patterns of plant roots in the soil, which is closely associated with nutrient uptake efficiency [30]. An excellent root architecture can actively respond to N nutrition signals to explore and absorb available N in the soil [31]. Studies have shown that plants extend root length and increase root surface area to anchor the plant, enhance water and nutrient acquisition, and improve crop productivity under LN stress [32]. In the present study, tall fescue increased both the longest root length and total root length under LN stress, suggesting a root foraging response that may increase the potential for NO_3_^−^ acquisition.

Nitrogen is a key component of photosynthetic enzymes and pigments [26]. N absorbed by plants and allocated to leaves is primarily used for the biosynthesis of photosynthetic components, including Chl, C assimilation enzymes, and coenzymes [33]. Negi et al. reported that LN stress disrupts chloroplast structure in plant cells, leading to a reduction in Chl content and increasing plant susceptibility to photodamage in *Chlorella sorokinian* [34]. LN-induced photoinhibition impairs the photochemical efficiency of photosystem II [35]. Therefore, Chl content and F_v_/F_m_ displayed a significant decline due to LN in this study, which in turn caused a decrease in P_n_ in tall fescue (Figure 4). Furthermore, our correlation analysis across both LN and MN treatments revealed that shoot N content and biomass were strongly and positively correlated with Chl content (*r* = 0.997 and 0.991, respectively), Pn (*r* = 0.931 and 0.874), and F_v_/F_m_ (*r* = 0.976 and 0.963) (Table S3). These strong correlations suggest that the decline in plant N status induced by LN is tightly coupled with the reduction in photosynthetic capacity, which collectively restricts biomass accumulation in tall fescue [36].

To further investigate the molecular basis of LN stress responses in tall fescue, we performed RNA-seq of leaves after 14 days of LN treatment. GO enrichment analysis showed that DEGs were enriched in terms such as C fixation, phosphoenolpyruvate carboxykinase activity, and phosphoenolpyruvate carboxylase activity (Figure 8A). KEGG enrichment analysis showed that DEGs were enriched in pathways such as C fixation by the Calvin cycle and N metabolism (Figure 8B). Collectively, these results suggest that LN induces transcriptional reprogramming of genes involved in both N and C metabolism in tall fescue.

Nitrogen metabolism mainly includes N uptake, transport, and assimilation [37]. Plant N uptake capacity is reflected by N absorption efficiency, while N assimilation capacity is primarily reflected by N utilization efficiency [38]. N absorption and utilization efficiencies play crucial roles in plant adaptation to LN stress [39]. NRT1 is typically involved in long-distance nitrate transport and signal perception, whereas NRT2, in association with its partner protein NAR2, constitutes a high-affinity transport system responsible for efficient nitrate uptake under LN stress [40,41]. In line with these functional roles, our study showed that the expression of nitrate-transport-related genes, *NRT1* and *NRT2.4*, was significantly upregulated (Figure 9B), suggesting that tall fescue activated both low- and high-affinity nitrate uptake systems in response to LN stress [42]. This upregulation is characteristic of the LN stress response. NIGT1/HRS1 repressors are downregulated under LN stress, relieving NRT2 inhibition and maximizing NO_3_^−^ scavenging [43].

Nitrogen assimilation is the process by which plants convert inorganic N (NO_3_^−^ and NH_4_^+^) into organic nitrogenous compounds via a series of redox reactions [16]. NO_3_^−^ absorbed by roots is first reduced to NO_2_^−^ by NR in the cytoplasm and then transported to plastids and further reduced to NH_4_^+^ by NiR [13]. The ammonium is subsequently incorporated into organic forms through the GS/GOGAT cycle [44]. NR serves as the rate-limiting enzyme in this pathway, and the reduction in NR activity impairs the conversion of NO_3_^−^ to NO_2_^−^ and downstream amino acid synthesis, thereby compromising organic N utilization [13]. As NR and NiR are nitrate-inducible genes, LN relieves their nitrate-dependent transcriptional induction, which is consistent with their marked downregulation observed in this study (Figure 9B) [45]. In contrast, the significant upregulation of GS suggests a metabolic shift from primary nitrate assimilation toward internal N recycling, in which ammonium released from protein degradation and photorespiration is reassimilated through GS [46,47]. This is consistent with the decreased Chl content observed in tall fescue under LN (Figure 9B). Taken together, these results indicate that LN suppressed nitrate reduction. The lower NR activity would be expected to reduce the demand for C skeletons and reducing power during nitrate assimilation, which may contribute to soluble sugar accumulation and the elevated C/N ratio.

Besides N metabolism, efficient coordination between C fixation and downstream metabolism is essential for plant growth and development [48]. To mitigate the damage caused by LN stress, plants undergo adaptive responses, including reduced biomass accumulation, altered shoot and root growth, and decreased sink demand [49]. These changes result in excess C accumulation in source tissues such as leaves and leaf sheaths. In maize, fructose accumulation could reach up to three-fold relative to control [50], and a similar trend was observed in our study, as indicated by the increase in soluble sugars, including fructose, glucose, and sucrose, in tall fescue shoots (Figure 6A–C). The accumulation of soluble sugars is also reported to be a survival strategy of plants grown under LN stress [51]. This might be the reason why the content of soluble sugars in roots is also significantly higher under LN conditions than under MN conditions (Figure 6D–F). Furthermore, our findings showed that several genes associated with C metabolism, particularly those involved in glycolysis and the TCA cycle, responded to LN stress. In the glycolysis pathway, *GAPDH1* and *PEPC1* are upregulated, while *GAPDH2* and *PEPC2* are downregulated by LN stress (Figure 9A). Glycolysis converts glucose to pyruvate, simultaneously producing ATP and NADH [51]. Downregulation of this pathway would restrict the supply of respiratory substrate and reduce energy production, thereby suppressing plant growth [52]. Glyceraldehyde-3-phosphate dehydrogenase (GAPDH) is a key enzyme of glycolysis, responsible for catalyzing the conversion of glyceraldehyde-3-phosphate (G3P) to 1,3-bisphosphoglycerate [53]. Previous studies have shown that cadmium stress suppressed GAPDH activity and consequently reduced G3P synthesis, resulting in a decline in sucrose biosynthesis and alteration in C partitioning. These changes inhibited plant growth and biomass accumulation in pepper (*Capsicum annuum*) [54]. Studies have shown that phosphoenolpyruvate carboxylase (PEPC) modulates the TCA cycle and glycolysis, thereby altering the levels of intermediates and downstream metabolites [55]. The TCA cycle supports energy production and N assimilation through the supply of C skeletons [56]. In this study, the key genes of the TCA cycle, including *ME*, *MDH*, and *OGDH*, were generally downregulated in response to LN stress (Figure 9A). LN stress impairs N assimilation, reducing the demand for C skeletons from TCA cycle intermediates, which may be associated with a lower TCA cycle flux [57]. Meanwhile, the growth restriction reduces C sink demand, while sustained photosynthetic C fixation generates excess carbohydrates that cannot be utilized for structural synthesis, leading to substantial soluble sugar accumulation [49]. Collectively, these results suggested that LN stress remodels the transcription of key glycolytic and TCA-cycle genes in tall fescue.

Transcription factors are proteins that regulate gene expression by binding to specific DNA sequences. They are crucial for cell development, differentiation, and homeostasis in the regulatory network of plants [58]. In this study, 27 TFs were identified as having significantly differential expression under LN stress, belonging to seven distinct families, namely bHLH, HSF, ERF, GATA, MYB, MADS, and AP2 (Figure 10). Among them, the bHLH TF family was the most abundant, followed by the HSF TF family (Figure 10B). Some bHLH TFs have previously been reported to regulate various responses to LN stress. Yang et al. discovered that overexpression of *TabHLH1* significantly improved tolerance to N deprivation, as indicated by larger plant phenotype, greater biomass, and higher N concentration and accumulation than the wild type under N-starvation conditions [59]. *MhbHLH130* improved nitrogen uptake efficiency in apple (*Malus hupehensis*) rootstock by enhancing flavonoid biosynthesis and reducing lignin accumulation [60]. In contrast, the role of HSF TFs in regulating LN tolerance has been rarely reported, with only a single study documented. The nitrogen sensor *NLP3* enhances heat tolerance in rice by regulating the heat shock factors *HsfA3*/*HsfA7* and also increases nitrogen content in shoots and roots [61]. Therefore, HSF TFs are speculated to act as downstream regulators of LN tolerance rather than directly regulating it. Our study revealed that various TFs exhibited distinct expression patterns under LN stress, potentially reflecting the adaptive regulatory strategies of plants across different time points (Figure 11). Continuously upregulated genes may play a crucial regulatory role in the long-term response to LN stress, while early upregulated genes may participate in the early perception and rapid response to LN stress [62]. In contrast, continuously downregulated genes may negatively regulate plant LN tolerance. The expression of *FaILR3* was initially upregulated and then gradually declined with prolonged LN stress (Figure 11G,H), a pattern similar to the expression patterns of the reported nitrogen regulators *ZmbHLH118* and *TabHLH1* [59,63]. And previous studies have shown that *MdILR3* can directly bind to the promoters of *MdNRT2.3/2.4* and *MdNIA1*, thereby promoting nitrate uptake and assimilation [64]. Similarly to the expression pattern of the reported nitrogen regulator *MdORG2* [65], *FaIRO2* and *FabHLH69-P* were continuously upregulated throughout 48 h of LN stress (Figure 11A–C). In contrast, *FabHLH062* was continuously downregulated during 48 h of LN stress (Figure 11E,F), similar to the expression pattern of the reported nitrogen regulator *MdbHLH130* [60]. Based on the above results, we speculate that *FaIRO2*, *FabHLH69-P*, and *FabHLH062* may be candidate LN-response genes, and their functions and regulatory mechanisms under LN stress will be validated and elucidated in future studies.

Integrating the physiological and transcriptomic results, we propose a mechanistic model of the LN response in tall fescue. LN stress is first perceived in the root, where NO_3_^−^ transporter and assimilation genes were upregulated (Figure 9B). In the shoot, the downregulation of genes related to photosynthesis paralleled the declines in Chl, Pn and Fv/Fm (Figure 4). Meanwhile, reduced NR activity suggested suppressed NO_3_^−^ reduction, which would be expected to reduce the consumption of C skeletons and reductant, potentially leading to soluble sugar accumulation and an elevated C/N ratio (Figure 6A–C). The resulting C surplus may be allocated to roots, as indicated by increased root soluble sugars, and may potentially support root architectural plasticity under LN (Figure 6D–F). Upregulation of NO_3_^−^ transporter and GS genes suggested an increased potential for root N acquisition and internal N recycling, which may partially alleviate N starvation, potentially forming an adaptive feedback loop.

## 4. Materials and Methods

### 4.1. Plant Materials and Growth Conditions

Tall fescue (*F. arundinacea* cv. ‘K31’) seeds were germinated in culture dishes with moistened filter paper. After the seeds germinated and developed shoots and roots, the seedlings were transferred to black hydroponic boxes (127 × 87 × 114 mm) containing half-strength Hoagland solution. Seedlings were placed in a growth chamber (XBQH-1, Xubang, Jinan, Shandong province, China) for a 21-day culture period. The growth chamber was programmed with a 14/10 h day/night cycle, a day/night temperature of 25/20 °C, a relative humidity of 75%, and a photosynthetically active radiation of 600 µmol·m^−2^·s^−1^.

### 4.2. Experimental Treatments

In this study, LN and MN treatments were established in a hydroponic system using a modified Hoagland solution. NO_3_^−^ was the sole nitrogen source, and its concentration was adjusted to 0.25 mM and 4 mM, respectively [66]. The composition of the MN solution was as follows: 1.5 mM KNO_3_, 1.25 mM Ca(NO_3_)_2_, 7 mM K_2_SO_4_, 2 mM Mg_2_SO_4_, 125 µM KH_2_PO_4_, 0.03 mM Fe-EDTA, 0.3 µM CuSO_4_·5H_2_O, 0.77 µM ZnSO_4_·7H_2_O, 9.2 µM MnCl_4_·4H_2_O, 46 µM H_3_BO_3_, 0.4 µM Na_2_MoO_4_. The composition of the LN solution was as follows: 0.25 mM KNO_3_, 1.25 mM CaCl_2_, 14.625 mM K_2_SO_4_, 2 mM Mg_2_SO_4_, 125 µM KH_2_PO_4_, 0.03 mM Fe-EDTA, 0.3 µM CuSO_4_·5H_2_O, 0.77 µM ZnSO_4_·7H_2_O, 9.2 µM MnCl_4_·4H_2_O, 46 µM H_3_BO_3_, 0.4 µM Na_2_MoO_4_. The seedlings were wrapped with sponge and inserted into holes in a polystyrene board, which was floated on the nutrient solution containing different nitrate concentrations in plastic containers (20 cm in diameter and 22 cm in depth), with 15 plants per container. The nutrient solution was replaced weekly and adjusted to pH 6.0. Other environmental conditions were maintained as described above. Each container was treated as one biological replicate, and plants within the same container were pooled or sampled representatively. Three experiments were performed independently under identical growth conditions.

Experiment 1: 21-day-old seedlings were subjected to LN and MN treatments for 14 d, with three independent containers used as biological replicates (*n* = 3) for RNA-seq.

Experiment 2: 21-day-old seedlings were subjected to LN and MN treatments for 35 d. At 35 d after treatment, phenotypic and physiological parameters were measured. Four independent containers were used as four biological replicates (*n* = 4).

Experiment 3: 21-day-old seedlings were subjected to LN and MN treatments under the same conditions. Three independent containers per treatment were used as biological replicates (*n* = 3) for temporal expression patterns of TFs.

### 4.3. Measurements of Plant Growth Indexes

Growth-related indicators, including shoot height, leaf blade length, leaf sheath length, and the longest root length, were measured using a ruler [67,68]. Plants were cut at the stem base, and shoot biomass was determined with an analytical balance. Root biomass was determined by drying to a final constant weight at 65 °C [29].

### 4.4. Root Morphology Analysis

The whole root system was divided into crown roots and lateral roots. Total root length, total root volume, total root surface area, and total root number were defined as the sum of the corresponding parameters of lateral roots and crown roots [69]. To quantify the length, number, surface area and volume of crown roots and lateral roots, the entire root system of individual plants was rinsed with tap water. The roots were placed in a transparent container filled with distilled water and scanned with an EPSON 1680 scanner (Seiko Epson Corporation, Beijing, China). The scanned images were analyzed using root imaging analysis software (WinRHIZO Pro version 2013b, Regent Instruments Inc., Quebec City, QC, Canada) [67].

### 4.5. Determination of Total Chl Content, P_n_ and Fv/Fm

For total Chl content, 0.1 g of fresh leaves was taken and immediately soaked in 6 mL dimethyl sulfoxide at room temperature in darkness for the extraction. The absorbance at 665 and 649 nm was measured with a spectrophotometer (Ultrospec 2100 pro, Biochrom Ltd., Cambridge, UK). After extraction, the leaves were dried at 80 °C to constant weight. The Chl content was determined based on calculations by Zheng et al. [70].

The P_n_ of leaves was measured using a LCproT (ADC Bioscientific Ltd. Hoddesdon, UK). The leaves were clamped into the leaf chamber, where the CO_2_ concentration was set to 425 ppm, the red-blue light source was set to 500 mmol photo·m^−2^·s^−1^, and the leaf temperature was maintained at 25 °C.

The F_v_/F_m_ was measured using an IMAGING-PAM (Heinz Walz, Effeltrich, Germany). The second fully expanded leaves were dark-adapted and then placed on the detection stage. The leaf clip was closed, and the detection stage was covered with a light-shielding cloth. Detection and recording were performed using the ImagingWin software (Heinz Walz GmbH, Effeltrich, Germany).

### 4.6. Determination of NO_3_^−^ Concentration, N Content and NR Activity

The concentration of NO_3_^−^ in tall fescue was detected using the salicylic acid–sulfuric acid solution reported by Hachiya and Okamoto [71]. The shoots and roots of the plants were dried and ground using a grinder. An accurate weight of 80 mg of dry sample was mixed with 10 volumes of deionized water preheated to 80 °C, and incubated at 100 °C for 20 min. A 30 µL aliquot of the supernatant was mixed with 120 µL of 0.05% (*w*/*v*) salicylic acid–sulfuric acid solution, vortexed, and incubated at 25 °C for 20 min. Then, 3 mL of 8% (*w*/*v*) NaOH was added. The absorbance at 410 nm was measured with a spectrophotometer (Ultrospec 2100 pro, Biochrom Ltd., Cambridge, UK). The NO_3_^−^ concentration was determined by a standard curve. The standard curve was established with 0, 0.125, 0.25, 0.5, 1, 2, 4, and 8 mM potassium nitrate following the same procedure.

Nitrogen content in shoots and roots was determined by the Kjeldahl method [72]. An aliquot of 0.2 g dry sample was accurately weighed to the nearest 0.0001 g using an analytical balance, wrapped in filter paper, and placed into digestion tubes. Then, 10 mL of concentrated sulfuric acid and catalyst were added to each tube. Digestion was performed in a Foss digestion unit at 420 °C until the solution became clear. After digestion, the samples were sequentially analyzed using a Kjeldahl nitrogen analyzer.

Nitrate reductase activity was determined following the method described by Gao et al. [73]. At 35 d after treatment, 0.5 g of fresh leaves was homogenized with 5 mL of extraction buffer (1.211 g cysteine and 0.372 g EDTA dissolved in 1 L phosphate buffer, pH 8.7) and then centrifuged. The supernatant was collected as the crude enzyme extract. A 1 mL measure of crude enzyme extract was incubated with 0.4 mL of 0.2% (*w*/*v*) NADH and 1.2 mL of 0.1 M KNO_3_ (dissolved in phosphate buffer, pH 7.5) in a water bath at 25 °C for 1 h. The reaction without NADH solution was used as the blank control. The reaction was then terminated by adding 1 mL of 1% sulfanilamide, followed by adding 1 mL of 0.02% N-(1-naphthyl) ethylenediamine dihydrochloride. After 1 h of incubation, the samples were centrifuged at 4000 rpm for 15 min, and the absorbance of the supernatant was measured at 540 nm. NR activity was expressed as μmol NO_2_^−^ g^−1^ FW h^−1^. One unit of NR activity was defined as the amount of enzyme that produced 1 μmol NO_2_^−^ per hour per gram fresh weight.

### 4.7. Determination of Soluble Sugar Content

Soluble sugar content was determined using an improved phenol-sulfuric acid method described by Liu et al. [74]. After treatment, dried samples (0.05 g) were extracted with 10 mL of absolute alcohol and incubated in a water bath at 30 °C for 30 min, then centrifuged at 6800 rpm for 10 min. The mixture of 1 mL supernatant, 5 mL of 98% concentrated sulfuric acid and 1 mL of 23% phenol was incubated in a water bath at 30 °C for 30 min. Absorbance was then measured at 490 nm using a spectrophotometer (Ultrospec 2100 pro, Biochrom Ltd., Cambridge, UK). The contents of glucose, sucrose, and fructose were quantified using respective standard curves. A blank control was prepared with 1 mL of 23% phenol, 1 mL of 80% ethanol, and 5 mL of 98% concentrated sulfuric acid.

### 4.8. Transcriptomic Analysis

At 14 d after treatment, approximately 0.2 g of leaves were collected for RNA-seq. Three independent biological replicates were used for each treatment group (*n* = 3). The total RNA samples from tall fescue leaves were extracted using an RNAprep Pure Plant Kit (TIANGEN, Beijing, China), their integrity was assessed using the Agilent 2100 bioanalyzer (Agilent Technologies, Santa Clara, CA, USA), and all samples had RIN values ≥ 6. The cDNA libraries were prepared using standard library construction and sequenced on the Illumina sequencing platform by Novogene (Beijing, China). This study used fastp (0.19.7) to filter the raw data and thus obtained clean reads. After that, StringTie was used to reassemble the transcriptome [75]. HISAT (2.0.5) was used to align the reads to the tall fescue ‘K31’ genome (PRJNA1312083) [76,77]. Differential expression analysis was conducted using DESeq2. Genes with |log_2_ fold change| ≥ 1 and adjusted *p*-value (*P*adj) ≤ 0.05 were defined as DEGs. Gene Ontology (GO) enrichment analysis and Kyoto Encyclopedia of Genes and Genomes (KEGG) pathway enrichment analysis were performed using GOseq (v1.10.0) and KOBAS (v2.0.12), based on the identified DEGs. In addition, differential expression transcription factors were screened from the DEGs using a stricter threshold |log_2_ fold change| ≥ 2 and *P*adj ≤ 0.05.

### 4.9. Validation of the Temporal Expression Patterns of TFs in LN Stress by qRT-PCR

Samples of approximately 0.05 g leaves were collected at 0, 1, 3, 6, 12, and 48 h after treatment. The total RNA was extracted using the SPARKeasy Plant RNA Rapid Extraction Kit (Sparkjade, Shandong, China), following the manufacturer’s protocol. A 1 μg measure of RNA was used in the ABScript III RT Master Mix for qPCR with gDNA Remover (ABclonal, Wuhan, China) reverse transcription. qRT-PCR was performed using BrightCycle Blue Universal SYBR Green qPCR Mix with UDG (ABclonal, Wuhan, China). The qRT-PCR was run in a two-step protocol, and melt curve analysis was performed using the default program of the Roche LightCycler 480 II machine (Roche Diagnostics, Rotkreuz, Switzerland). Relative expression levels were determined using *FaActin7* as the reference gene [78]. Specific primers are listed in Table S2. Gene expression was quantified using the 2^−ΔΔCT^ method [79].

Tall fescue is an allohexaploid species whose genome comprises three subgenomes, designated G1, G2, and P. To distinguish homoeologous gene copies located on different subgenomes, we adopted a nomenclature in which the subgenome origin is indicated by a suffix after the gene symbol, separated by a hyphen. For example, *FaIRO2-G1* and *FaIRO2-P* represent the *FaIRO2* copies located on the G1 and P subgenomes, respectively [68].

### 4.10. Validation of DEGs by Quantitative Reverse Transcription Polymerase Chain Reaction (qRT-PCR)

Six C-N metabolism genes and three TFs, namely *FaNR (LoarK31hap1_130343)*, *FaGS (LoarK31hap1_80432)*, *FaNRT2.4 (LoarK31hap1_52067)*, *FaME (LoarK31hap1_1507)*, *FaMDH (LoarK31hap1_130421)*, *FaOGDH (LoarK31hap1_19757)*, *FaLBD37 (LoarK31hap1_67811)*, *FaNFYA7 (LoarK31hap1_67162)*, and *FaERF1 (LoarK31hap1_142338)*, were selected for analysis by qRT-PCR.

### 4.11. Statistical Analysis

Statistical analysis was performed using SPSS 26 software (SPSS Inc., Chicago, IL, USA). Student’s *t*-test was employed for pairwise comparisons between the LN and MN treatments. Duncan’s test was used to compare the expression levels of each transcription factor across time points under LN stress. Pearson correlation analysis was performed using pooled data from both LN and MN treatments (*n* = 6) to evaluate relationships among Chl content, Pn, Fv/Fm, shoot N content, and shoot biomass. Statistical significance was set at *p* < 0.05, 0.01, and 0.001. All data are expressed as means ± standard error. Graphical representations were generated using GraphPad Prism 10.1.2 (GraphPad Software Inc., La Jolla, CA, USA).

## 5. Conclusions

In summary, LN stress severely inhibited shoot growth, altered root architecture, reduced photosynthetic performance, and weakened N metabolic capacity while increasing the content of soluble sugars in tall fescue. Transcriptomic analysis revealed that LN stress triggered coordinated alterations in both C and N metabolic pathways, specifically affecting genes involved in N transport, N assimilation, glycolysis, and the TCA cycle. Finally, we focused on the expression patterns of differentially expressed TFs under LN stress and identified three candidate genes potentially involved in the LN stress response in tall fescue. Collectively, this study provides integrated physiological and transcriptional insights into tall fescue responses to LN stress and highlights potential target genes for genetic improvement of perennial forage and turf grasses with high N use efficiency.

## Data Availability

The raw sequence data reported in this paper have been deposited in the NCBI sequence read archive database (BioProject ID: PRJNA1538747). The original contributions presented in this study are included in the Supplementary Material. Further inquiries can be directed to the corresponding author.

## Supplementary Materials

The following supporting information can be downloaded at https://www.mdpi.com/article/10.3390/plants15213261/s1. Figure S1: Transcriptomic analysis of LN and MN samples. A. Principal component analysis (PCA) based on gene expression value (FPKM). Each point represents one biological replicate (*n* = 3). B. Pearson correlation analysis between samples; Table S1: Quality control metrics for raw sequencing data; Table S2: Primers used in this study; Table S3: Significant correlation of shoot nitrogen content and shoot biomass with total chlorophyll content, net photosynthetic rate and photochemical efficiency.
