# Supplementary material for: Integrated Physiological and Transcriptomic Profiling of Tall Fescue in Response to Low-Nitrogen Stress
Source: Plants (Basel). 2026 Oct 22;15(21):3261. doi: 10.3390/plants15213261 (PMC13765978; doi:10.3390/plants15213261)
Supplement: Supplementary file 1 [file plants-15-03261-s001.zip › plants-4602778-supplementary.pdf]

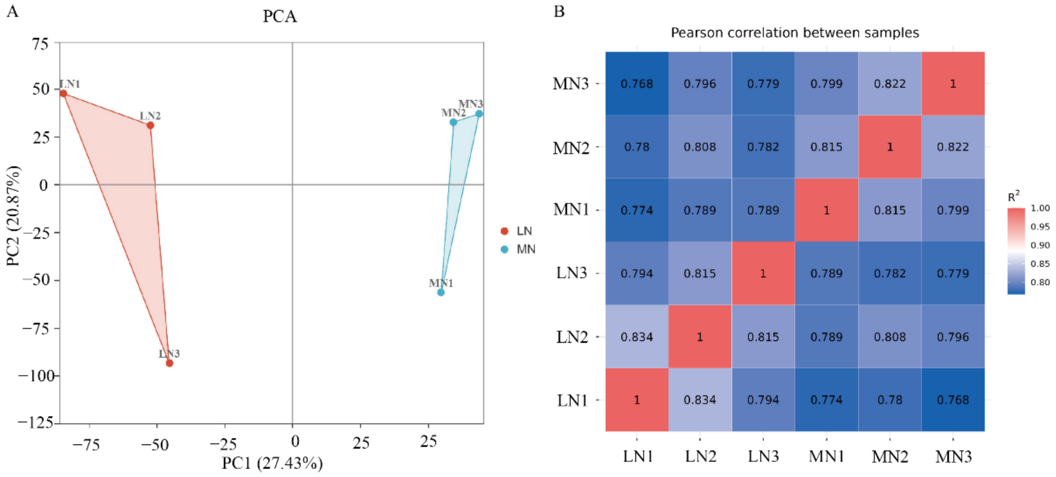

Figure S1. Transcriptomic analysis of LN and MN samples. A. Principal component analysis (PCA) based on gene expression value (FPKM). Each point represents one biological replicate (*n* = 3). B. Pearson correlation analysis between samples.

Table S1. Quality control metrics for raw sequencing data.

sample	library	raw_reads	raw_bases	clean_reads	clean_bases	error_rate	Q20	Q30	GC_pct
LN1	FRAS260317407-1r	44111404	6.62G	42504524	6.38G	0.01	99.4	97.41	52.86
LN2	FRAS260317408-2r	44449824	6.67G	43420124	6.51G	0.01	99.39	97.18	52.39
LN3	FRAS260317409-1r	44446980	6.67G	42848650	6.43G	0.01	99.42	97.4	53.67
MN1	FRAS260317410-1r	44072760	6.61G	42081724	6.31G	0.01	99.4	97.4	53.37
MN2	FRAS260317411-1r	45709480	6.86G	43864752	6.58G	0.01	99.46	97.55	53.49
MN3	FRAS260317412-1r	44133142	6.62G	42275780	6.34G	0.01	99.44	97.48	53.93

Table S2. Primers used in this study.

Primer name	Forward primer (5′-3′)	Reverse primer (5′-3′)
FaActin7	AGATCAAGGTCGTTGCTCCA	CTCCCAGACTAGACGATACAGC
LoarK31hap1_55972	TCAACGGGCTCTACTCTTCC	ATGCTGAGAACTCCTGGCTT
LoarK31hap1_63968	CCGATCACACCAAGAAGCTG	TCTAGGGAAGTTGCGGAGAC
LoarK31hap1_61802	CCTAGGAACCGCGAACAGTA	CCAGGGCTGTTTCCGTAATG
LoarK31hap1_53238	GGAAACCCTAGGAACCACGA	CAGTCTCGTTGCATTTCCGT
LoarK31hap1_77912	CTACTTCCTCGGGACATGCT	TGATGCTCTTGGAAGGCTGA
LoarK31hap1_86809	CCAGCTACTTCCTTGGGACA	TGATGCTCTTGGAAGGCTGA
LoarK31hap1_4466	GGGCTCCGAGTTCATCTACA	ATCCCAGTATCACGCTGGAG
LoarK31hap1_17202	GGGCTCCGAGTTCATCTACA	TGGACGGGATCTCTTCTTGG
LoarK31hap1_95453	TGATGAACACGATGCTCCAG	CAGGTAGTCCTGTTGGTGGT
LoarK31hap1_14767	GTCCAATGTGCCTCCATCAC	CTCTCTTCCATGGCTTGCAC
LoarK31hap1_40001	ACCATCTCCTGGAACGAGTC	CCGACCTTCCTGAATCCGTA
LoarK31hap1_75145	AAACACGGAGAGCTTCTGGA	GTTGAGGTGGTACAGGCTCT
LoarK31hap1_96349	AGCACTCGTGGCCGATATAC	AACCACACCTCGAGTTGGAA
LoarK31hap1_66548	TATGGCTATTCGCCGTCCTC	CGTACGTCTTGCTCAGGAAC
LoarK31hap1_130343	GGAGATCGGTCTGGTCTTCG	GTGGTGTTCAGGAATGGCGT
LoarK31hap1_80432	CTTGTCTTTGGGTGGTGGGT	GCTTATAGCCAACAACCGGC
LoarK31hap1_52067	CCGACGAGGTTGGAAAGCGT	CCCATCCTGCCGACCAGAAC
LoarK31hap1_1507	CGATCGACGACTGCCGTAAG	TCATGCTCGTGAGCGAATGG
LoarK31hap1_130421	CTCTTGAAGCCGGTGATGGC	GGGACGACACCACTTGGAAC
LoarK31hap1_19757	AGCACCCACTCCATCCTAGC	GACCTTGGCTCGGCTTTCTC

LoarK31hap1_67811	CCGTCTTCGTCGCCAAGTTC	CGCCTCGTACAGAAGCGACC
LoarK31hap1_67162	AAGCCGTGGCTCATTTCCAG	TCTCCAGTTCAGCCTTTGCG
LoarK31hap1_142338	TGCAGAGGGAAGCTCATCCT	ATGTCGTCGTTGTCGTCGTC

Table S3. Significant correlation of shoot nitrogen content and shoot biomass with total chlorophyll content, net photosynthetic rate and photochemical efficiency.

	Total chlorophyll content	Net photosynthetic rate	Photochemical efficiency
Correlation with shoot nitrogen content			
Pearson *r*	0.997	0.931	0.976
P value	<0.001***	0.007**	0.001**
R^2	0.994	0.867	0.953
Correlation with shoot biomass			
Pearson *r*	0.991	0.874	0.963
P value	<0.000***	0.023*	0.002**
R^2	0.982	0.764	0.927

Note: Pearson correlation analysis was performed using pooled data from both LN and MN treatments (n = 6). Asterisks indicate significance levels: *$P < 0.05$, **$P < 0.01$, ***$P < 0.001$. R^2 represents the coefficient of determination.
